# Initial Metabolic Profiles Are Associated with 7-Day Survival among Infants Born at 22-25 Weeks of Gestation

**DOI:** 10.1016/j.jpeds.2018.03.032

**Published:** 2018-07

**Authors:** Scott P. Oltman, Elizabeth E. Rogers, Rebecca J. Baer, James G. Anderson, Martina A. Steurer, Matthew S. Pantell, J. Colin Partridge, Larry Rand, Kelli K. Ryckman, Laura L. Jelliffe-Pawlowski

**Affiliations:** 1 Department of Epidemiology and Biostatistics and the Preterm Birth Initiative, University of California San Francisco, San Francisco, CA; 2 Department of Pediatrics, University of California San Francisco, San Francisco, CA; 3 Preterm Birth Initiative, University of California San Francisco, San Francisco, CA; 4 Department of Pediatrics, University of California San Diego, La Jolla, CA; 5 Department of Epidemiology and Biostatistics and Pediatrics, University of California San Francisco, San Francisco, CA; 6 Preterm Birth Initiative, Department of Obstetrics, Gynecology, and Reproductive Sciences, University of California San Francisco, San Francisco, CA; 7 Department of Epidemiology and Pediatrics, University of Iowa, Iowa City, IA

**Keywords:** AUC, Area under the curve, IVH, Intraventricular hemorrhage, NBS, Newborn screening, NRN, Neonatal Research Network, ROC, Receiver operating curve, TPN, Total parenteral nutrition

## Abstract

**Objective:**

To evaluate the association between early metabolic profiles combined with infant characteristics and survival past 7 days of age in infants born at 22-25 weeks of gestation.

**Study design:**

This nested case-control consisted of 465 singleton live births in California from 2005 to 2011 at 22-25 weeks of gestation. All infants had newborn metabolic screening data available. Data included linked birth certificate and mother and infant hospital discharge records. Mortality was derived from linked death certificates and death discharge information. Each death within 7 days was matched to 4 surviving controls by gestational age and birth weight z score category, leaving 93 cases and 372 controls. The association between explanatory variables and 7-day survival was modeled via stepwise logistic regression. Infant characteristics, 42 metabolites, and 12 metabolite ratios were considered for model inclusion. Model performance was assessed via area under the curve.

**Results:**

The final model included 1 characteristic and 11 metabolites. The model demonstrated a strong association between metabolic patterns and infant survival (area under the curve [AUC] 0.885, 95% CI 0.851-0.920). Furthermore, a model with just the selected metabolites performed better (AUC 0.879, 95% CI 0.841-0.916) than a model with multiple clinical characteristics (AUC 0.685, 95% CI 0.627-0.742).

**Conclusions:**

Use of metabolomics significantly strengthens the association with 7-day survival in infants born extremely premature. Physicians may be able to use metabolic profiles at birth to refine mortality risks and inform postnatal counseling for infants born at <26 weeks of gestation.

Recent improvements in neonatal resuscitation and intensive care have led to an increase in survival among infants born at 22-25 weeks of gestation.^1^, ^2^, ^3^ These neonates suffer high rates of mortality and morbidity, especially at lower gestational ages and birth weights.^4^, ^5^, ^6^, ^7^, ^8^, ^9^ This makes decisions about resuscitation and use of active interventions difficult for clinicians and families.^2^, ^10^, ^11^, ^12^, ^13^ Compounding the magnitude of clinical decisions regarding infants born extremely preterm is the fact that up to 83% of deaths in infants born at 22-24 weeks of gestation occur in the first week of life.^5^

Given the uncertainty surrounding clinical management of periviable newborns, the American Academy of Pediatrics recommends that decisions regarding care should be individualized and family centered, taking into account conditions and risk factors known to affect outcomes.^14^ There are several models to predict survival in this patient population, with the most widely used model being the National Institute of Child Health & Human Development Neonatal Research Network (NRN) Extremely Preterm Birth Outcome calculator, by Tyson et al.^15^ However, all of these predictive models rely exclusively on clinical characteristics of the infant.^15^, ^16^, ^17^

The use of unique sources of data and novel possible predictors may improve survival prediction for infants born at <26 weeks of gestation. One such opportunity is the use of metabolic data measured as part of newborn screening (NBS) for rare inborn errors of metabolism.^18^ Our group and others have shown that infants born at term and preterm are metabolically distinct and that metabolic profiles can be used for accurate gestational dating.^19^, ^20^, ^21^, ^22^, ^23^ Furthermore, metabolites measured as part of routine NBS have been implicated in neonatal complications, including respiratory distress syndrome, patent ductus arteriosus, and necrotizing enterocolitis.^24^, ^25^

The objective of this study was to assess the relationship between survival to 7 days of age and metabolic markers in concert with common infant characteristics. We hypothesized that the incorporation of metabolites would strengthen the association with survival beyond the capacity of infant characteristics alone.

## Methods

This was a nested, case-control study within a retrospective cohort of data collected by the California Office of Statewide Health Planning and Development on births from 2005 to 2011 in California. This database contains information from hospital discharge, birth certificate, and death records from birth to 1 year of age. This database was combined with results from NBS using date of birth, hospital of birth, birth weight, and birth time. In California, the Newborn Screening Program (administered by the California Department of Public Health) screens all newborn infants for rare inborn metabolic diseases by measuring markers in a heel-stick blood spot taken between 12 hours and 8 days after birth. The program has been described extensively elsewhere.^26^, ^27^

**Figure 1:**
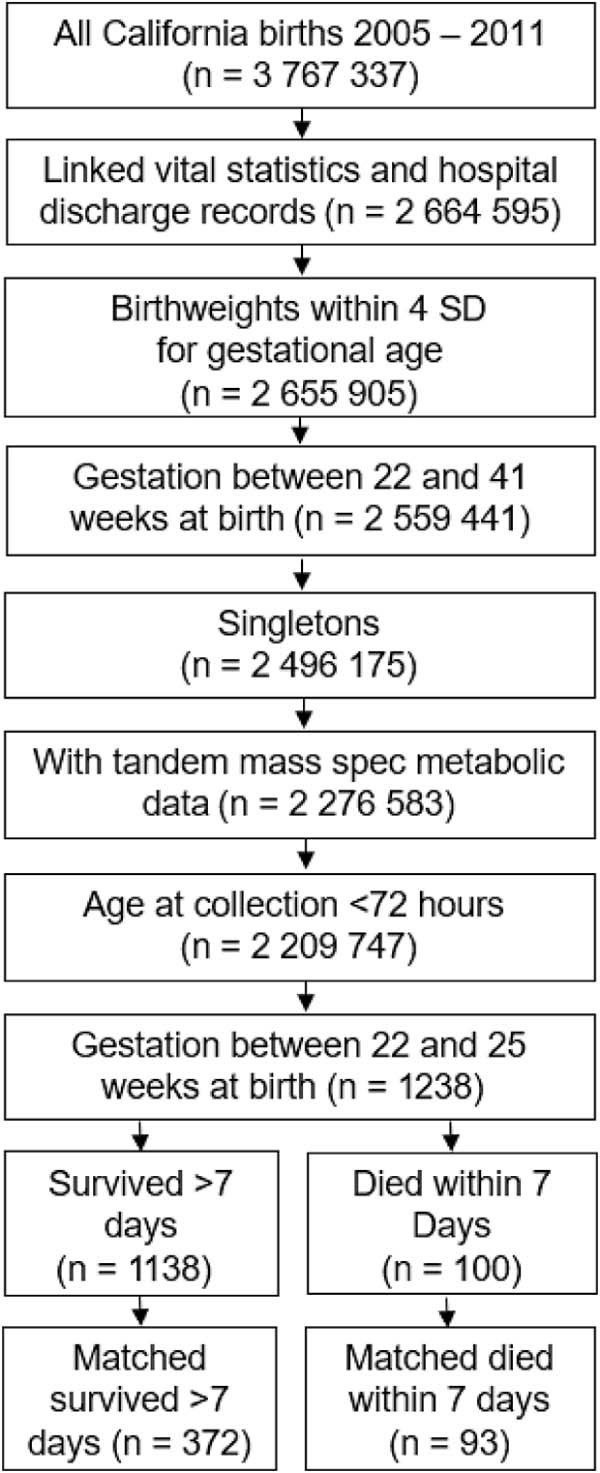
Flow chart of infants born in California between 2005 and 2011 eligible for analysis.

There were 2 664 595 infants in the source cohort after linkage with vital statistics. Infants were excluded if the birth weight was >4 SDs from the mean for gestational age by sex (to limit potential erroneous data),^28^ they were not singletons, they did not have complete NBS metabolic data (this includes infants who died after delivery but before their sample could be analyzed), or if their NBS blood spot was not taken within 72 hours of delivery (Figure 1; available at www.jpeds.com). The population was then limited to infants born at <26 weeks of gestation, leaving 1238 infants eligible for analysis. From the final population, infants who died within 7 days were matched to 4 controls by gestational week of birth and birth weight z-score category, which left a final sample of 93 cases and 372 controls (7 infants who died could not be matched to a control).

The outcome of interest for this study was survival past 7 days of age. Henceforth, “survival” in our study will be defined as alive after 7 days and “death” will be defined as deceased at <7 days. Mortality data were obtained from death certificate and death discharge information within the California Office of Statewide Health Planning and Development dataset. Infant and maternal characteristics used in analyses included birth weight, gestational age, sex, delivery type, maternal education, race/ethnicity, Medi-Cal status (California's Medicaid), use of total parenteral nutrition (TPN), birth weight small for gestational age, and any intraventricular hemorrhage (IVH) diagnosis (*International Classification of Diseases, Ninth Revision, Clinical Modification* codes 772.13, 772.14). Criteria for choosing these specific variables included availability, occurrence before NBS, and previous evidence of an association with early infant mortality. Administration of TPN was obtained from the NBS data and was defined as receiving TPN before the blood spot was taken. There were 42 metabolites entered into the model consisting of 12 amino acids, 26 acylcarnitines, free carnitine, 2 hormones, and 1 enzyme (Table I; available at www.jpeds.com). In addition, 12 metabolic ratios were considered in the model. We used the same ratios created by NBS to detect known inborn errors of metabolism. No additional ratios were created.

Standardized mass spectrometry (tandem mass spectrometry) was used to measure the amino acids, acylcarnitines, and free carnitine. Thyroid-stimulating hormone and 17-hydroxyprogesterone were measured by high-performance liquid chromatography, and galactose-1-phosphate uridyltransferase was measured with a fluorometric enzyme assay.^26^

### Statistical Analyses

All metabolites underwent a natural log transformation before being used in analyses to minimize the skew and influence of outlying observations. Birth weight was transformed into z scores using standardized growth curves^28^ and then divided into 10 categories by increments of 0.5. Birth weight small for gestational age was calculated for infants with birth weight <10th percentile from standardized growth curves.^28^ Population characteristics of interest and metabolites were summarized by the use of means with SDs and frequencies with proportions for continuous and categorical variables, respectively. Univariate analyses used *t* tests and χ^2^ tests for continuous and categorical variables, respectively, to compare infants that survived and those that did not.

Multivariable logistic regression was used to model the association with survival. We used a stepwise regression procedure that consecutively adds explanatory variables based on minimizing *P* value from univariable analyses and then removes any variables with newly recalculated *P* values that eclipse the predetermined threshold. To maximize model performance, all possible explanatory variables had the potential to enter the model, and the criteria for remaining in the model was *P* < .10. In addition, variables with a *P* ≥ .05 and < .10 were removed if their inclusion raised the Akaike information criterion or had no discernible positive effect on area under the curve (AUC) from a receiver operating characteristic (ROC) curve. If both base metabolites and their ratio were selected for inclusion in the model, severe multicollinearity was assessed and the components were evaluated independently with *P* values and contribution to model AUC and Akaike information criterion. Age at NBS collection and TPN were entered into the model build, as both are well-known to influence values of NBS metabolites,^29^, ^30^, ^31^ and if not selected, a model forcing them in was compared with the original model. In addition, the model was examined in subgroups stratified by TPN and by IVH to compare predictive performance and metabolites of interest.

Performance of the final model was evaluated with AUC, and variables were summarized via OR, 95% CI, and standardized parameter estimates. Cross-validation was applied to assess the model fit, and the final model underwent conditional logistic regression to test for bias due to matching. The final model also was compared (using AUC, 95% CIs, and model contrast tests) to models consisting of metabolites only and clinical characteristics only (sex, IVH diagnosis, race/ethnicity, maternal education, Medi-Cal status, and cesarean delivery). ROC-derived probability cut points were used to calculate sensitivity and specificity, and odds of survival above particular cut points were compared with that of the referent cut point (<0.3) to generate ORs with 95% CIs.

All analyses were performed with SAS 9.3 (SAS institute, Cary, North Carolina). Methods and protocols for the study were approved by the Committee for the Protection of Human Subjects within the Health and Human Services Agency of the State of California and the institutional review board of the University of California, San Francisco.

## Results

The matched study sample included 93 infants who died and 372 survivors from a retrospective source population of 1238 infants born in California at 22-25 weeks of gestation from 2005 to 2011 with completed NBS. Within the source population, 100 (8.1%) infants died at <7 days, and 305 (24.6%) died within the first year with an average age of survival of 35.0 days. Each infant who died at <7 days was matched to 4 infants surviving >7 days of the same gestational age week and approximate birth weight. In the matched sample, variables that differed significantly between infants that survived and those that did not were TPN administration, IVH diagnosis, and age at NBS collection (Table II). In univariable analysis of the metabolites, 3 enzymes and hormones, 3 amino acids, 14 acylcarnitines, and 9 metabolic ratios differed significantly between infants who survived and those who did not (Table I).

**Table II:** Maternal demographics and infant characteristics in the matched sample and population sample

Demographics	Matched			All		
	Survived past 7 days	Did not survive 7 days	*P* value	Survived past 7 days	Did not survive 7 days	*P* value
	n = 372	n = 93		n = 1138	n = 100	
Gestational age, wk	23.9 (0.8)	23.9 (0.8)	1.00	24.3 (0.8)	23.9 (0.8)	<.001
Birth weight, g	668 (111.4)	673.7 (117.9)	.662	722.2 (149.2)	662.9 (124.7)	<.001
Age at collection, h	37.5 (16.6)	30.2 (14.3)	<.001	36.5 (16.3)	30.2 (14.3)	<.001
Female	186 (50.0)	39 (41.9)	.164	538 (47.3)	42 (42.0)	.311
TPN	284 (76.3)	61 (65.6)	.034	873 (76.7)	66 (66.0)	.016
Small for GA	10 (2.7)	2 (2.2)	.770	57 (5.0)	6 (6.0)	.665
Cesarean delivery	202 (54.3)	64 (68.8)	.011	684 (60.1)	70 (70.0)	.052
Medi-Cal	197 (53.0)	52 (55.9)	.609	626 (50.0)	57 (57.0)	.701
IVH	175 (47.0)	66 (71.0)	<.001	498 (43.8)	68 (68.0)	<.001
Race			.140			.093
White	57 (15.3)	14 (15.1)		163 (14.3)	16 (16.0)	
Black	40 (10.8)	3 (3.2)		121 (10.6)	3 (3.0)	
Hispanic	207 (55.6)	55 (59.1)		631 (55.4)	59 (59.0)	
Asian	42 (11.3)	10 (10.8)		134 (11.8)	10 (10.0)	
Other	26 (7.0)	11 (11.8)		89 (7.8)	12 (12.0)	
Maternal education			.879			.891
<12 y	128 (34.4)	31 (33.3)		394 (34.6)	33 (33.0)	
12 y	89 (23.9)	26 (28.0)		281 (24.7)	28 (28.0)	
>12 y	133 (35.8)	31 (33.3)		411 (36.1)	34 (34.0)	
Unknown	22 (5.9)	5 (5.4)		52 (4.6)	5 (5.0)	

*GA*, gestational age.

Continuous variables described using mean and SD and categorical variables using frequencies and proportions. *T* tests and χ^2^ tests for continuous and categorical variables respectively were used to compare cases and controls.

The model most strongly associated with survival was adjusted for age at collection and contained 12 variables: IVH diagnosis, alanine, ornithine, C-3, C-3DC, C-5OH, C-6, C-10:1, C-12:1, C-14:1, C-16, and C-16:1 and had an area under the ROC curve (AUC) of 0.885 (95% CI 0.851-0.920). Of all the variables in the model, the 2 amino acids alanine and ornithine were most robustly linked to survival with standardized estimates of –0.60 and 0.52, respectively (Table III).

**Table III:** Multivariable logistic regression model to predict survival past 7 days

Predictors	Survived past 7 days (n = 372)*	Did not survive 7 days (n = 93)*	Parameter estimate	OR (95% CI)	Model *P* value	Standardized estimate
IVH	175 (47.0)	66 (71.0)	−0.92	0.40 (0.21-0.75)	.005	−0.25
Age at collection	37.52 (16.6)	30.20 (14.3)	0.02	1.02 (1.00-1.05)	.049	0.21
Alanine	5.36 (0.41)	5.59 (0.68)	−2.26	0.11 (.05-0.24)	<.001	−0.60
C-3	0.90 (0.4)	0.63 (0.5)	0.90	2.47 (1.08-5.62)	.032	0.22
C-3DC	−2.40 (0.50)	−2.43 (0.48)	−0.82	0.44 (0.22-0.88)	.020	−0.22
C-5OH	−1.52 (0.43)	−1.77 (0.57)	0.91	2.49 (1.27-4.90)	.008	0.24
C-6	−2.97 (0.8)	2.81 (0.8)	−0.45	0.64 (0.41-1.00)	.048	−0.20
C-10:1	−2.96 (0.96)	−3.27 (1.07)	0.54	1.72 (1.20-2.45)	.003	0.29
C-12:1	−3.54 (0.9)	−3.45 (0.9)	−0.40	0.67 (0.45-1.01)	.055	−0.19
C-14:1	−2.35 (0.54)	−2.23 (0.66)	−1.32	0.27 (0.14-0.52)	<.001	−0.41
C-16	0.13 (0.38)	−0.15 (0.39)	1.59	4.92 (1.86-130)	.001	0.35
C-16:1	−2.06 (0.5)	−2.25 (0.6)	0.71	2.03 (1.05-3.94)	.036	0.22
Ornithine	4.65 (0.5)	4.36 (0.6)	1.82	6.14 (2.82-13.41)	<.001	0.52

All metabolites are natural log transformed.

* Frequency (%) and mean (SD) for categorical and continuous predictors, respectively.

**Figure 2:**
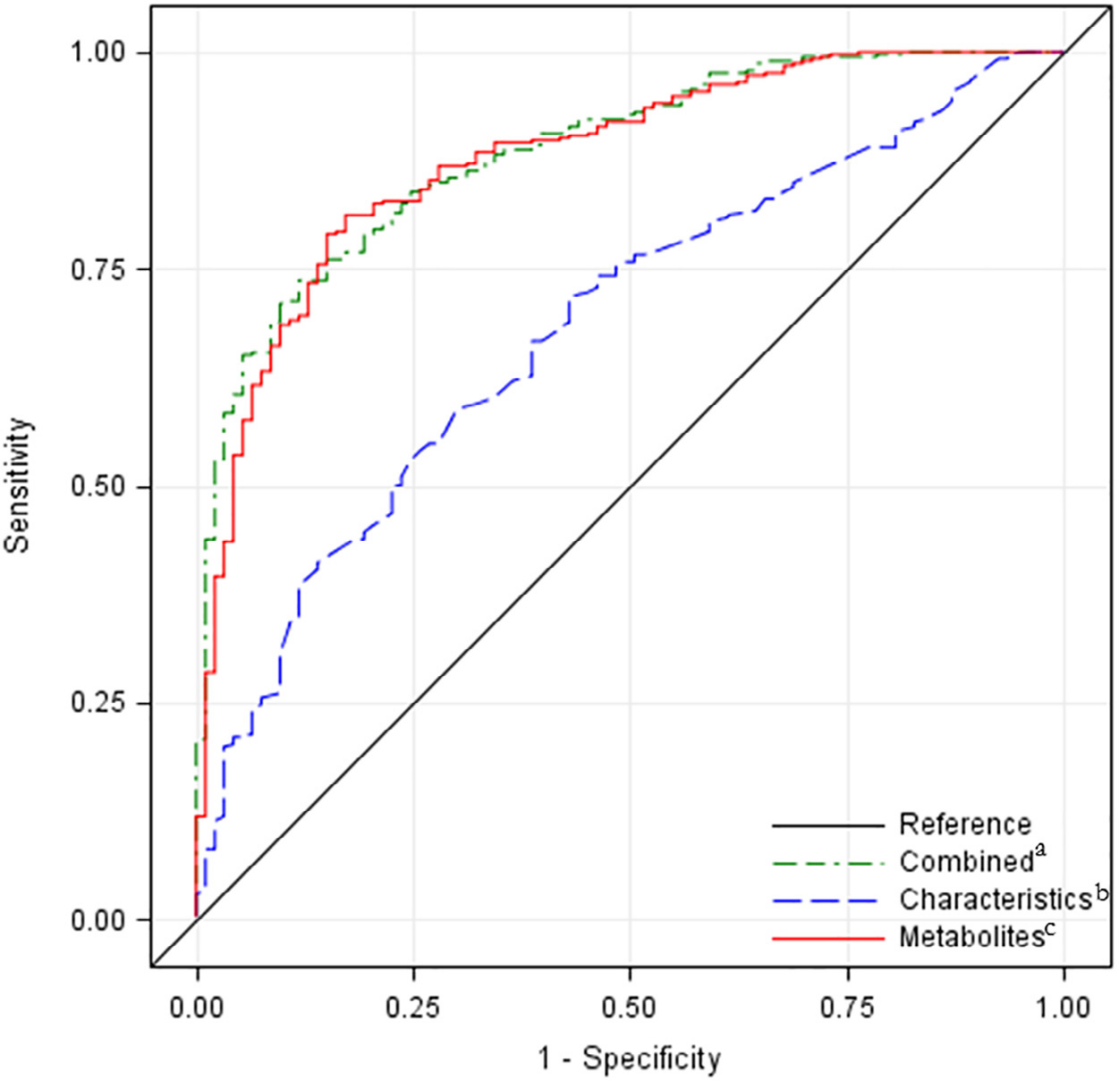
Receiver operating curves for three different models of survival. ^a^Combined model (AUC 0.885, 95% CI 0.851-0.920) adjusted for age at collection consists of IVH diagnosis, alanine, C-3, C-3DC, C-5OH, C-6, C-10:1, C12:1, C14:1, C-16, C-16:1, and ornithine. ^b^Characteristics only model (AUC 0.685, 95% CI 0.627-0.742) consists of sex, IVH diagnosis, race/ethnicity, maternal education, Medi-Cal status, and caesarean delivery. ^c^Metabolites-only model (AUC 0.879, 95% CI 0.841-0.916) limited to metabolites from the final combined model adjusted for age at collection.

The model maintained strong performance after cross-validation (AUC 0.857, 95% CI 0.817-0.896). The full model also performed better when compared with a metabolites-only model (AUC 0.879, 95% CI 0.841-0.916, *P* = .278) or a clinical characteristics-based model (AUC 0.685, 95% CI=0.627-0.742, *P* < .001; Figure 2). After conditional logistic regression was applied to the full model, there was minimal evidence of bias due to matching (results not shown). The model remained robust when adjusted or stratified by TPN and when stratified by IVH. In fact, the model exhibited stronger performance in infants without TPN administered before NBS (AUC 0.935 vs 0.867) and in infants without IVH (AUC 0.918 vs 0.869; Table IV; available at www.jpeds.com).

Association with survival was evaluated with the use of probability cut points generated from the ROC curve. Of the 245 infants with a probability score ≥0.9, 240 (98.0%) survived. These 240 infants also accounted for 64.5% of all infants who ended up surviving, and they were 360 times more likely (OR 360.0, 95% CI 91.6-1414.6) to survive when compared with infants with a probability score <0.3. Alternatively, no infants with probability scores <0.1 survived. This cut point successfully discriminated the mortality of 15 (16.13%) infants without misclassifying a survivor (Table V). When classification was expanded to the full population, the positive predictive value at a probability score of 0.9 was 99.2%, and the negative predictive value at a probability score of 0.1 was 75.0% (Table V).

**Table V:** Performance of the final survival model at various probability cut points

	Survived past 7 days (n)	Did not survive 7 days (n)	OR (95% CI)	Sensitivity (95% CI)	Specificity (95% CI)	PPV* (95% CI)	NPV* (95% CI)
Samples	372	93					
≥0.9	240	5	360.0† (91.6-1414.6)	64.5 (59.4-69.4)	94.6 (87.9-98.2)	99.2 (98.2-99.7)	18.5 (15.3-22.2)
≥0.8	296	19	116.8† (37.3-366.0)	79.6 (75.1-83.6)	79.6 (70.0-87.2)	97.8 (96.6-98.7)	24.8 (20.2-29.9)
≥0.7	321	31	77.6† (25.7-234.8)	86.3 (82.4-89.6)	66.7 (56.1-76.1)	96.8 (95.6-97.8)	29.7 (23.9-36.1)
≥0.6	342	41	62.6† (21.0-186.5)	91.9 (88.7-94.5)	55.9 (45.2-66.2)	96.0 (94.7-97.1)	35.6 (28.2-43.6)
≥0.5	354	52	51.1† (17.3-150.8)	95.2 (92.5-97.1)	44.1 (33.8-54.8)	95.0 (93.5-96.2)	36.7 (28.1-46.0)
≥0.4	364	59	46.3† (15.7-136.1)	97.9 (95.8-99.1)	36.6 (26.8-47.2)	94.4 (93.1-95.8)	41.9 (31.3-53.0)
≥0.3	368	63	43.8† (14.9-128.6)	98.9 (97.3-99.7)	32.3 (22.9-42.8)	94.2 (92.7-95.5)	48.5 (36.0-61.1)
<0.3	4	30	Reference				
<0.1	0	15	—	100.0 (99.0-100.0)	16.1 (9.3-25.2)	93.0 (91.4-94.4	75.0 (50.9-91.3)

*NPV*, negative predictive value; *PPV*, positive predictive value.

* PPV and NPV calculated using the entire cohort.

† *P* value <.001.

## Discussion

Using metabolic markers that are collected as a part of routine NBS, we were able to successfully build an algorithm that reliably classified infants who survived past 7 days of age when born at 22-25 weeks of gestation. The final model consisted of IVH diagnosis and 11 metabolites (9 acylcarnitines and 2 amino acids).

Past predictive models have exclusively used maternal and infant characteristics to predict survival.^15^, ^16^, ^17^ Although the majority of infant mortality in neonates born extremely preterm occurs within the first 24 hours after birth, another significant portion occurs within the first week of life, and accurate prediction is crucial for clinicians to appropriately counsel families through difficult decisions about on-going aggressive interventions.^5^, ^32^, ^33^ Currently, the most widely used predictive model is the NRN's Web-based outcomes calculator, which uses birth weight, gestational age, sex, exposure to antenatal corticosteroids, and singleton or multiple gestation (AUC 0.751, 95% CI 0.735-0.767).^15^ When comparing our model combining characteristics and metabolites to the model used for the NRN calculator, our model substantially improved performance. Furthermore, the metabolites alone markedly outperformed both the model used for the NRN calculator and our clinical characteristics-only model. The model used for the NRN calculator, however, performed better than our characteristics-only model. This is likely due to the fact that our model was matched on 2 of the NRN calculator's strongest variables (birth weight and gestational age) and the inclusion of antenatal corticosteroid exposure and singleton vs multiple gestation in the NRN calculator model. These results demonstrate that metabolic profiles may be of superior utility in the prognosis of survival for infants born extremely preterm, and with the addition of antenatal corticosteroid and gestation information, performance may be further improved. Moreover, metabolic profiles may point to etiologic pathways that could inform care.

Newborn metabolomics have been shown to be related to gestational age and birth weight, especially in infants born preterm.^20^, ^21^, ^23^ To minimize the influence of these factors on our model and to delineate independent metabolic profiles, we matched cases and controls by gestational age and birth weight z-score category. The success of the model under these circumstances suggests that infants who are less likely to survive have metabolic dysfunction that is unrelated to growth restriction or gestational age. This is further supported by the low proportion of birth weights that were small for gestational age among infants who did not survive in our sample. Because growth in utero relies on maternal glucose via placental transfer, the metabolic dysfunction in the infants who did not survive was possibly related to the inability to maintain independent glucose homeostasis.^34^, ^35^ Indeed, the 2 biggest correlates of survival in our model were relatively low concentrations of alanine and high concentrations of ornithine. Alanine is an important substrate in gluconeogenesis, and a build-up of alanine is emblematic of low enzymatic activity downstream.^36^ Ornithine, in contrast, plays a crucial role in cell proliferation, microvascularization, and kidney development, and the relatively high concentrations in neonates who survive are likely indicative of a properly functioning ornithine cycle.^37^ Further disruptions in glucose homeostasis can lead to abnormal concentrations of acylcarnitines, which are essential to beta-oxidation in the fetal liver.^34^, ^38^ These pathways of metabolic function may help explain how our model can be associated with survival using metabolic markers independently from birth weight and gestational age.

IVH was the only clinical diagnosis included as a variable in our model due to the fact that approximately 50% of cases occur on the first day of life and 90% by day 3,^39^, ^40^ which is the same time frame required for NBS sampling. Unsurprisingly, lack of an IVH diagnosis was associated with survival. This is supported in the literature, as mortality rates for IVH range from 4% for grade I to 40% for grade IV.^41^ Moreover, severe IVH in a neonate may lead to the withdrawal of intensive care measures.^42^ Given the complex relationship between IVH and survival, we examined the model in subsets of the sample as stratified by IVH. Regardless of IVH status, our model performed well, but unsurprisingly, the metabolites of importance varied between stratums. Specifically, the acylcarnitines C-5OH and C-14:1 were particularly important to survival in infants with IVH, suggesting they may be indicative of IVH severity and justifying further investigation.

Both age at NBS collection and administration of TPN were entered into our model because they are known to affect the concentrations of metabolites.^29^, ^30^, ^31^ Age at NBS remained in our model after selection, and a delay in collection was associated with increased survival. The most likely explanation for this is the positive correlation between probability of survival and infant age. TPN, however, failed to tolerate the selection process of the model build. As a result, we assessed the performance of our model after forcing in TPN and after stratifying the infant population based on TPN administration. The model displayed robust performance in all 3 scenarios, suggesting that TPN did not significantly alter the association with survival. It is important to note, however, that particular acylcarnitines had varying levels of importance between TPN stratums, warranting additional examination of the role TPN exposure plays within newborn metabolomics.

Our study had several strengths and limitations. Perhaps the most potent strength of our study is that our matched sample was derived from a population-based dataset that supports the potential generalizability of the model. Although we did not use separate training and validation datasets because of the very small number of babies born at very early gestations, we did use cross-validation as a means for ensuring our results were not due to overfitting. Our source data set was retrospective in nature, limiting us to the use of predetermined explanatory variables and outcome data. This was especially important as related to the NBS data. Infants who died before NBS could be obtained were not included in the model build. Although this limited our ability to examine survival no sooner than at 7 days and produced a smaller sample, we do not think it biased the results. We suspect that infants who died before NBS was completed would likely have had compromised metabolic functions to an even greater extent, and this may have improved the performance of our model. Nevertheless, future studies should assess metabolic function in infants who die within hours of birth. Given the retrospective data, we may not have been able to control for all factors affecting metabolic concentrations. Blood transfusions, for example, could have affected the values of metabolites, although, only in rare scenarios in which a transfusion occurs before NBS. An additional limitation of our data was the lack of information about antenatal corticosteroid exposure, which has been shown to be a powerful predictor of survival,^15^, ^43^, ^44^ and investigating the value of adding it to our model will be essential.

Our model should be validated in other clinical and population-based samples, preferably in a prospective setting. Validation of the model is essential to determine whether these metabolic patterns have predictive capacity. Moreover, the population of California that was used in this study is especially diverse, and the majority of infants in our study were of Hispanic ethnicity. Testing the model in other populations will help expand the generalizability of our results. In addition, our study focused on the association with survival at just one time point (7 days), and it may be that metabolic profiles are linked to survival at time-points beyond 7 days. Our study also focused on a single measurement of metabolic markers. To truly examine the probability of survival and elucidate the metabolic pathways that differentiate infants, measuring markers at multiple time points may be especially important. Furthermore, although NBS results currently require time for processing and interpretation that preclude them from being actively included in real-time decision-making, developing point of care testing is essential. Finally, given the value of metabolic correlates shown here, additional studies using them in the prediction of morbidities associated with preterm birth are warranted.

This study examined the link between routinely available metabolic markers and survival in extremely preterm newborns. We successfully built a model showing strong associations and demonstrated that infants who survived beyond 7 days were metabolically different from those infants who died in the first 7 days. Physicians potentially could use metabolic profiles measured shortly after birth to refine mortality risks and inform postnatal counseling for extremely premature infants.

## Figures and Tables

**Table I:** *T* tests comparing mean concentrations of individual metabolites between infants who survived ≥7 days and those who did not

Metabolites	Survived ≥7 days Mean (SD)	Did not survive >7 days Mean (SD)	*P* value
Enzymes and hormones			
17 Hydroxyprogesterone	5.08 (0.69)	5.24 (0.62)	.047
Thyroid-stimulating hormone	–0.41 (1.08)	−0.06 (0.79)	.004
Galactose-1-phosphate uridyltransferase	5.23 (0.30)	5.04 (0.35)	<.001
Amino acids			
5-Oxoproline	3.29 (0.67)	3.37 (0.57)	.292
Alanine	5.36 (0.41)	5.59 (0.68)	<.001
Arginine	2.89 (0.81)	2.88 (1.02)	.898
Citrulline	2.86 (0.45)	2.91 (0.53)	.391
Glycine	6.15 (0.34)	6.17 (0.42)	.619
Leucine/isoleucine	5.06 (0.48)	4.98 (0.72)	.228
Methionine	3.53 (0.88)	3.51 (1.07)	.854
Ornithine	4.65 (0.47)	4.36 (0.62)	<.001
Phenylalanine	4.32 (0.31)	4.33 (0.49)	.905
Proline	5.14 (0.54)	5.30 (0.77)	.020
Tyrosine	4.84 (0.81)	5.01 (0.86)	.064
Valine	5.04 (0.46)	4.95 (0.68)	.151
Amino acid ratios			
Arginine/ornithine ratio	−1.76 (0.68)	−1.49 (0.72)	.001
Citrulline/arginine ratio	−0.03 (0.75)	0.03 (0.79)	.431
Leucine/alanine ratio	–0.30 (0.39)	−0.60 (0.46)	<.001
Ornithine/citrulline ratio	1.79 (0.47)	1.45 (0.47)	<.001
Phenylalanine/tyrosine ratio	−0.51 (0.74)	−0.68 (0.60)	.041
Tyrosine/ornithine	0.18 (0.81)	0.65 (0.71)	<.001
Valine/phenylalanine ratio	0.71 (0.33)	0.62 (0.36)	.021
Carnitines			
Free carnitine	3.85 (0.43)	3.70 (0.47)	.003
C-2	3.16 (0.34)	3.03 (0.34)	.001
C-3	0.90 (0.43)	0.63 (0.47)	<.001
C-3DC	−2.40 (0.50)	−2.43 (0.48)	.710
C-4	−0.61 (0.41)	−0.72 (0.49)	.040
C-5	−0.78 (0.44)	−0.94 (0.51)	.002
C-5:1	−3.37 (0.74)	−3.41 (0.88)	.604
C-5DC	−2.07 (0.44)	−2.02 (0.39)	.350
C-5OH	−1.52 (0.43)	−1.77 (0.57)	<.001
C-6	−2.97 (0.81)	−2.81 (0.77)	.080
C-8	−2.60 (0.81)	−2.69 (0.87)	.367
C-8:1	−2.57 (0.90)	−2.79 (0.97)	.037
C-10	−3.13 (0.79)	−3.14 (0.80)	.969
C-10:1	−2.96 (0.96)	−3.27 (1.07)	.007
C-12	−2.78 (0.80)	−2.65 (0.69)	.146
C-12:1	−3.54 (0.86)	−3.45 (0.89)	.405
C-14	−1.99 (0.42)	−2.08 (0.43)	.048
C-14:1	−2.35 (0.54)	−2.23 (0.66)	.071
C-14OH	−4.07 (0.89)	−3.99 (0.78)	.452
C-16	0.13 (0.38)	−0.15 (0.39)	<.001
C-16:1	−2.06 (0.55)	−2.25 (0.62)	.004
C-16OH	−3.60 (0.87)	−3.65 (0.92)	.637
C-18	−0.45 (0.45)	−0.69 (0.39)	<.001
C-18:1	−0.18 (0.37)	−0.42 (0.41)	<.001
C-18:1OH	−3.97 (0.82)	−3.92 (0.78)	.598
C-18:2	−1.22 (0.77)	−1.56 (0.84)	<.001
C-18OH	−4.43 (0.79)	−4.34 (0.87)	.357
Carnitine ratios			
C-3/C-2 ratio	−2.26 (0.34)	−2.41 (0.36)	<.001
C-5/C-3 ratio	−1.68 (0.48)	−1.57 (0.52)	.054
C-5DC/C-3DC ratio	0.34 (0.34)	0.40 (0.37)	.090
C16OH/C16 ratio	−3.73 (0.87)	−3.51 (1.01)	.037
FC/(C16 + C18:1) ratio	3.16 (0.44)	3.27 (0.47)	.024

All metabolites are natural log transformed.

**Table IV:** Multivariable model performance with TPN forced in, stratified by TPN, and stratified by IVH

Predictors	Forced TPN (AUC 0.887)				Without TPN (AUC 0.935)				With TPN (AUC 0.867)			
	Survived past 7 days (n = 372)*	Did not survive 7 days (n = 93)*	OR (95% CI)	Model *P* value	Survived past 7 days (n = 88)*	Did not survive 7 days (n = 32)*	OR (95% CI)	Model *P* value	Survived past 7 days (n = 284)*	Did not survive 7 days (n = 61)*	OR (95% CI)	Model *P* value
IVH	175 (47.0)	66 (71.0)	0.40 (0.21-0.76)	.005	40 (45.5)	24 (75.0)	0.2 (.04-0.94)	.0415	135 (47.5)	42 (68.9)	0.52 (0.25-1.08)	.079
TPN	284 (76.3)	61 (65.6)	0.84 (0.41-1.73)	.634	–	–	–	–	–	–	–	–
Age at collection	37.52 (16.6)	30.20 (14.3)	1.03 (1.00-1.05)	.049	29.69 (14.5)	26.03 (11.7)	1.02 (0.94-1.09)	.678	39.95 (16.4)	32.39 (15)	1.02 (1.00-1.05)	.082
Alanine	5.36 (0.41)	5.59 (0.7)	0.10 (.05-0.24)	<.001	5.26 (0.4)	5.6 (0.8)	.06 (.01-0.33)	.001	5.39 (0.4)	5.58 (0.6)	0.12 (0.04-0.34)	<.001
C-3	0.90 (0.4)	0.63 (0.5)	2.46 (1.08-5.61)	.033	0.98 (0.4)	0.49 (0.4)	12.48 (0.98-159.4)	.052	0.88 (0.4)	0.7 (0.5)	1.56 (0.61-3.97)	.355
C-3DC	–2.40 (0.50)	–2.43 (0.5)	0.45 (0.23-0.91)	.026	–2.53 (0.5)	–2.63 (0.5)	0.35 (.06-2.17)	.261	–2.37 (0.5)	–2.32 (0.5)	0.43 (0.19-0.96)	.04
C-5OH	–1.52 (0.43)	–1.77 (0.6)	2.48 (1.25-4.94)	.009	–1.53 (0.5)	–1.75 (0.5)	1.88 (0.28-12.64)	.515	–1.51 (0.4)	–1.79 (0.6)	3.29 (1.19-9.05)	.021
C-6	–2.97 (0.8)	2.81 (0.8)	0.63 (0.41-0.99)	.045	–2.92 (0.8)	–2.83 (0.8)	0.52 (0.18-1.53)	.238	–2.99 (0.8)	–2.8 (0.8)	0.63 (0.37-1.06)	.083
C-10:1	–2.96 (0.96)	–3.27 (1.1)	1.72 (1.21-2.46)	.003	–3.24 (1.0)	–3.58 (1.0)	1.64 (0.77-3.49)	.196	–2.88 (0.9)	–3.11 (1.1)	1.74 (1.13-2.68)	.012
C-12:1	–3.54 (0.9)	–3.45 (0.9)	0.67 (0.45-1.01)	.055	–3.53 (0.9)	–3.58 (1.0)	0.51 (0.21-1.2)	.122	–3.54 (0.9)	–3.39 (0.8)	0.85 (0.51-1.39)	.507
C-14:1	–2.35 (0.54)	–2.23 (0.7)	0.27 (0.14-0.52)	<.001	–2.33 (0.6)	–2.25 (0.7)	0.16 (0.04-0.69)	.013	–2.35 (0.5)	–2.21 (0.6)	0.26 (0.11-0.6)	.002
C-16	0.13 (0.38)	–0.15 (0.4)	4.88 (1.84-12.9)	.001	0.2 (0.4)	–0.23 (0.4)	12.58 (1.21-130.56)	.034	0.12 (0.4)	–0.11 (0.3)	4.73 (1.58-14.14)	.005
C-16:1	–2.06 (0.5)	–2.25 (0.6)	2.03 (1.05-3.94)	.036	–1.98 (0.5)	–2.3 (0.6)	1.08 (0.17-6.7)	.934	–2.09 (0.6)	–2.23 (0.6)	2.26 (1.14-4.5)	.02
Ornithine	4.65 (0.5)	4.36 (0.6)	6.42 (2.88-14.30)	<.001	4.39 (0.4)	4.06 (0.5)	19.98 (1.73-231.21)	.017	4.73 (0.5)	4.51 (0.6)	5.15 (2.01-13.17)	<.001

	Without IVH (AUC 0.918)				With IVH (AUC 0.869)			
Predictors	Survived past 7 days (N = 197)*	Did not survive 7 days (N = 27)*	OR (95% CI)	Model *P* value	Survived past 7 days (N = 175)*	Did not survive 7 days (N = 66)*	OR (95% CI)	Model *P* value
IVH	—	—	—	—	—	—	—	—
TPN	—	—	—	—	—	—	—	—
Age at collection	38.41 (16.8)	29.63 (16.2)	1.03 (0.99-1.07)	.143	36.53 (16.3)	30.44 (13.5)	1.01 (0.98-1.05)	.474
Alanine	5.35 (0.4)	5.51 (0.7)	.04 (.01-0.26)	<.001	5.37 (0.4)	5.62 (0.7)	.09 (.03-0.26)	<.001
C-3	0.92 (0.4)	0.73 (0.5)	2.72 (0.55-13.41)	.219	0.88 (0.4)	0.59 (0.5)	1.98 (0.69-5.71)	.208
C-3DC	–2.43 (0.5)	–2.30 (0.5)	0.18 (.05-0.65)	.009	–2.38 (0.5)	–2.48 (0.4)	0.64 (0.27-1.54)	.322
C-5OH	–1.52 (0.5)	–1.71 (0.8)	1.53 (0.56-4.15)	.406	–1.51 (0.4)	–1.80 (0.4)	8.73 (2.09-36.47)	.003
C-6	–2.88 (0.7)	–2.67 (0.6)	0.38 (0.1-1.45)	.156	–3.08 (0.9)	–2.87 (0.8)	0.73 (0.44-1.2)	.213
C-10:1	–3.00 (1.1)	–3.54 (1.1)	2.05 (1.15-3.66)	.015	–2.92 (0.8)	–3.16 (1)	1.65 (0.95-2.85)	.075
C-12:1	–3.49 (0.8)	–3.24 (0.5)	0.33 (0.13-0.85)	.022	–3.59 (0.9)	–3.54 (1)	0.87 (0.53-1.43)	.592
C-14:1	–2.31 (0.5)	–2.21 (0.5)	0.46 (0.14-1.57)	.216	–2.39 (0.6)	–2.23 (0.7)	0.21 (0.08-0.52)	<.001
C-16	0.20 (0.4)	–0.09 (0.4)	6.98 (1.28-38.09)	.025	0.06 (0.4)	–0.18 (0.4)	4.18 (1.08-16.21)	.038
C-16:1	–2.04 (0.6)	–2.26 (0.8)	3.9 (1.39-10.91)	.01	–2.09 (0.5)	–2.25 (0.5)	1.46 (0.59-3.6)	.417
Ornithine	4.67 (0.5)	4.34 (0.6)	13.79 (2.78-68.46)	.001	4.64 (0.5)	4.37 (0.6)	6.49 (2.39-17.59)	<.001

All metabolites are natural log transformed.

* Frequency (%) and mean (SD) for categorical and continuous predictors, respectively.
